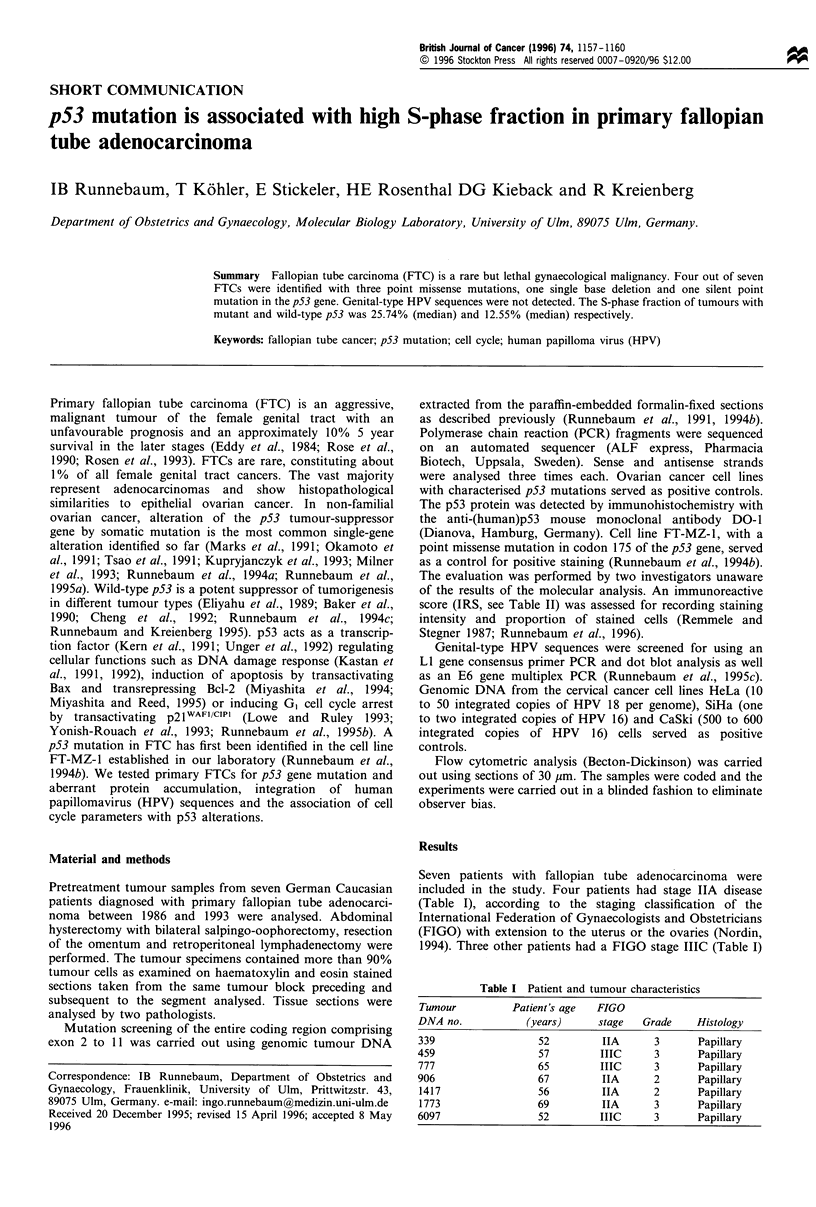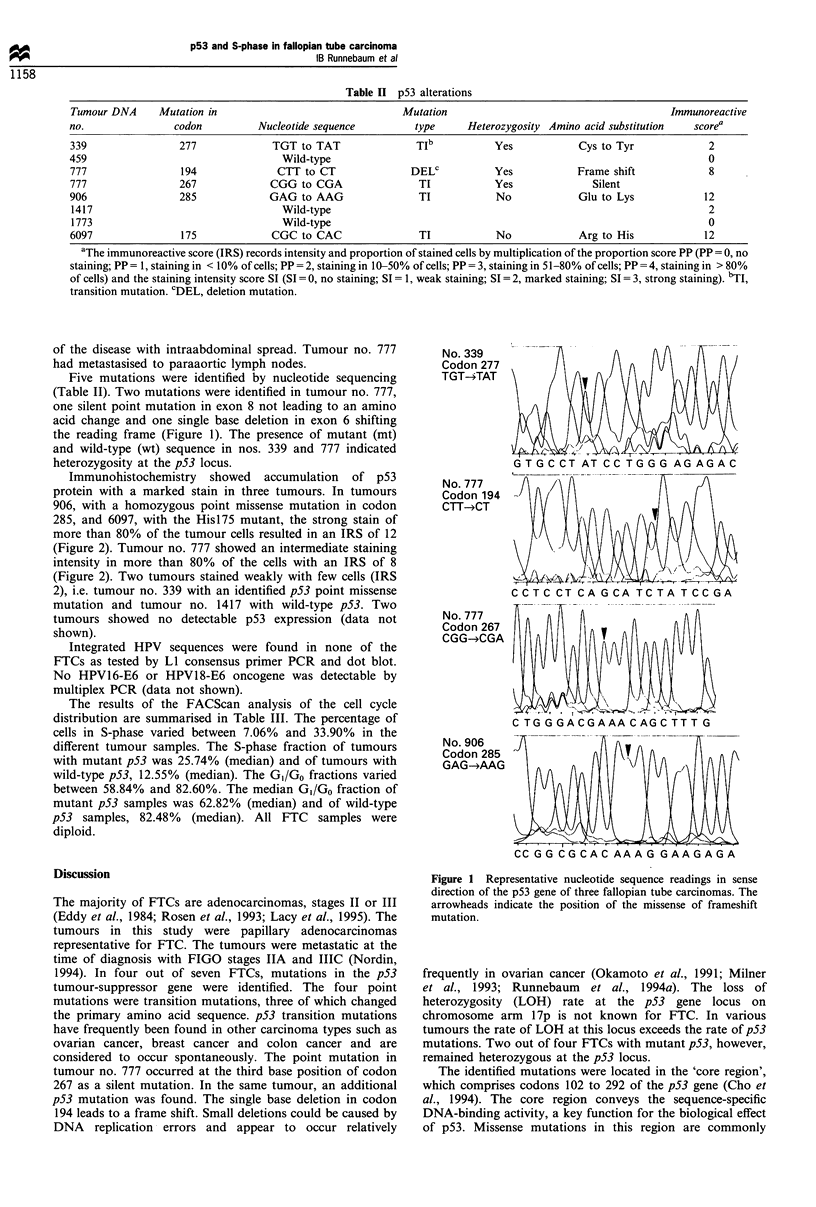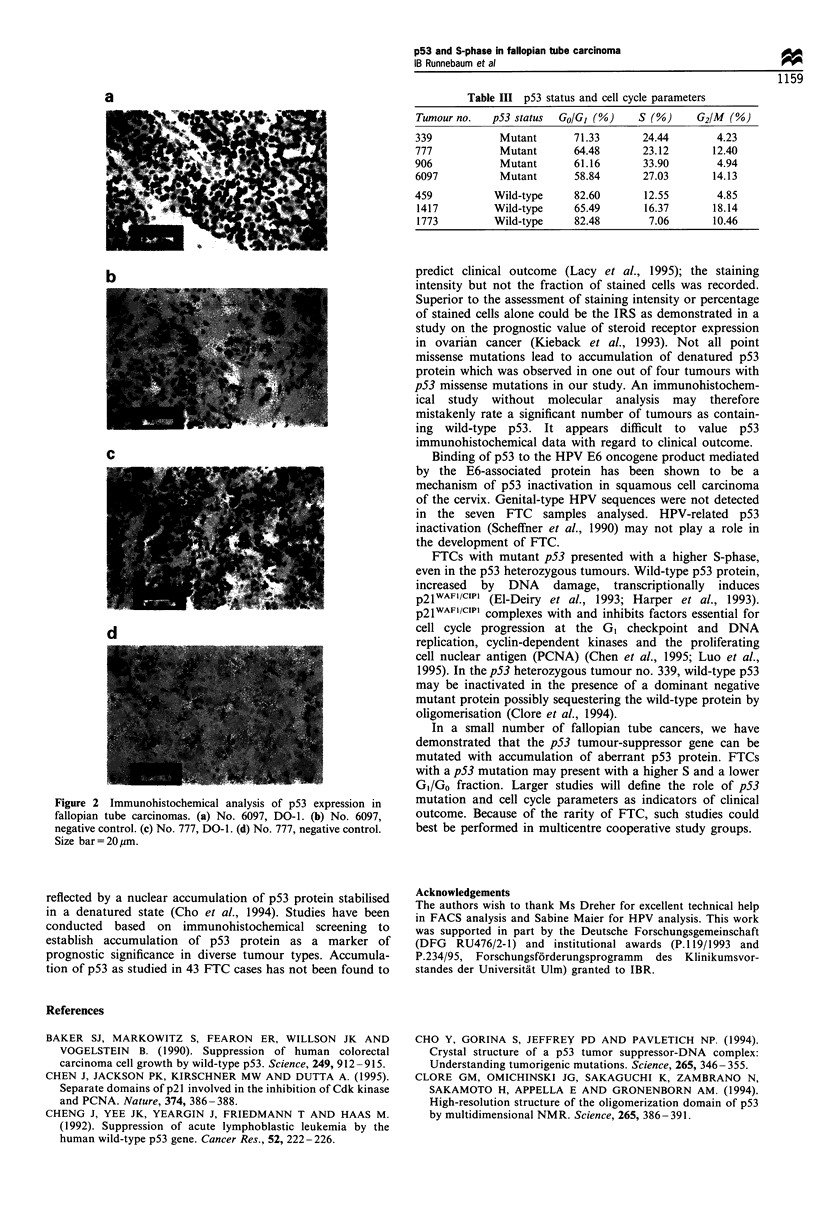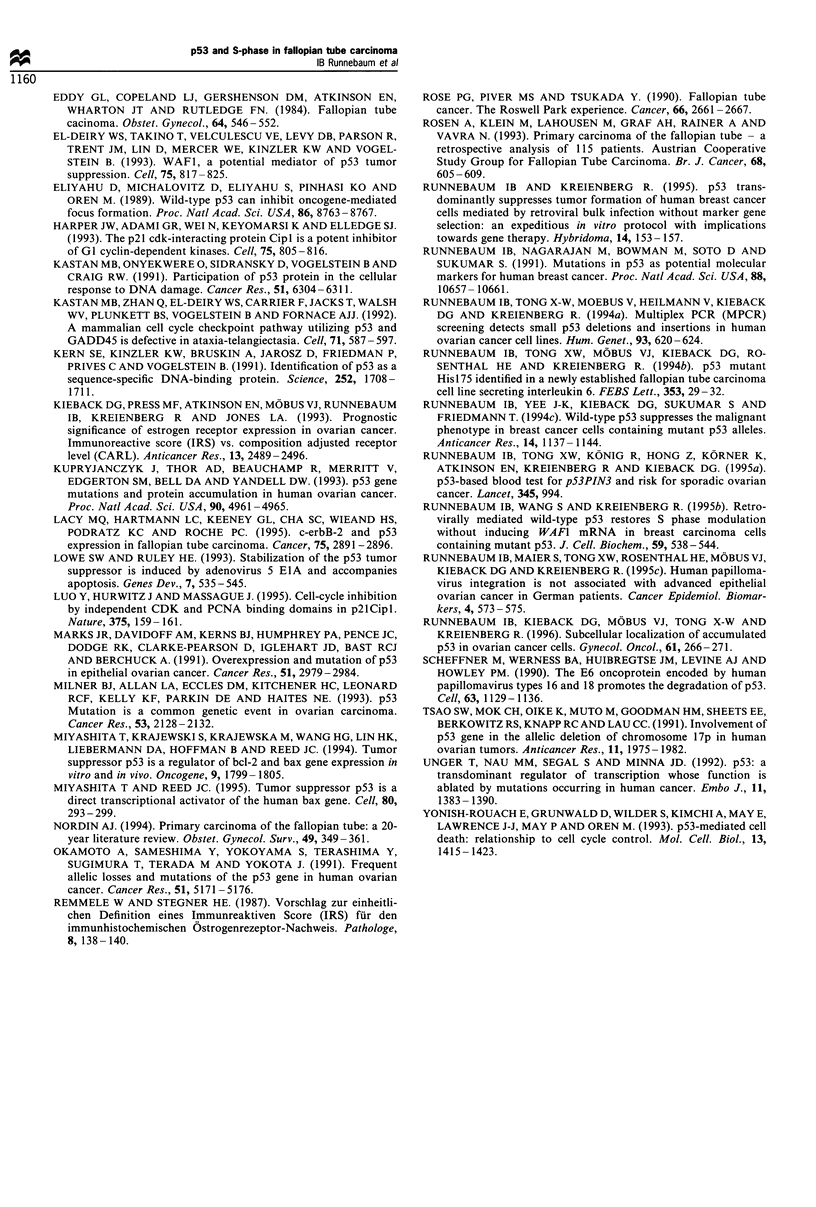# p53 mutation is associated with high S-phase fraction in primary fallopian tube adenocarcinoma.

**DOI:** 10.1038/bjc.1996.510

**Published:** 1996-10

**Authors:** I. B. Runnebaum, T. Köhler, E. Stickeler, H. R. Kieback, R. Kreienberg

**Affiliations:** Department of Obstetrics and Gynaecology, University of Ulm, Germany.

## Abstract

**Images:**


British Journal of Cancer (1996) 74, 1157-1160
© 1996 Stockton Press All rights reserved 0007-0920/96 $12.00

SHORT COMMUNICATION

### *p53* mutation is associated with high S-phase fraction in primary fallopian tube adenocarcinoma

IB Runnebaum, T Köhler, E Stickeler, HE Rosenthal DG Kieback and R Kreienberg

*Department of Obstetrics and Gynaecology, Molecular Biology Laboratory, University of Ulm, 89075 Ulm, Germany.*

**Summary** Fallopian tube carcinoma (FTC) is a rare but lethal gynaecological malignancy. Four out of seven FTCs were identified with three point missense mutations, one single base deletion and one silent point mutation in the *p53* gene. Genital-type HPV sequences were not detected. The S-phase fraction of tumours with mutant and wild-type *p53* was 25.74% (median) and 12.55% (median) respectively.

**Keywords:** fallopian tube cancer; *p53* mutation; cell cycle; human papilloma virus (HPV)

Primary fallopian tube carcinoma (FTC) is an aggressive, malignant tumour of the female genital tract with an unfavourable prognosis and an approximately 10% 5 year survival in the later stages (Eddy *et al.*, 1984; Rose *et al.*, 1990; Rosen *et al.*, 1993). FTCs are rare, constituting about 1% of all female genital tract cancers. The vast majority represent adenocarcinomas and show histopathological similarities to epithelial ovarian cancer. In non-familial ovarian cancer, alteration of the *p53* tumour-suppressor gene by somatic mutation is the most common single-gene alteration identified so far (Marks *et al.*, 1991; Okamoto *et al.*, 1991; Tsao *et al.*, 1991; Kupryjanczyk *et al.*, 1993; Milner *et al.*, 1993; Runnebaum *et al.*, 1994*a*; Runnebaum *et al.*, 1995*a*). Wild-type *p53* is a potent suppressor of tumorigenesis in different tumour types (Eliyahu *et al.*, 1989; Baker *et al.*, 1990; Cheng *et al.*, 1992; Runnebaum *et al.*, 1994*c*; Runnebaum and Kreienberg 1995). p53 acts as a transcription factor (Kern *et al.*, 1991; Unger *et al.*, 1992) regulating cellular functions such as DNA damage response (Kastan *et al.*, 1991, 1992), induction of apoptosis by transactivating Bax and transrepressing Bcl-2 (Miyashita *et al.*, 1994; Miyashita and Reed, 1995) or inducing $G_1$ cell cycle arrest by transactivating $p21^{WAF1/CIP1}$ (Lowe and Ruley 1993; Yonish-Rouach *et al.*, 1993; Runnebaum *et al.*, 1995*b*). A *p53* mutation in FTC has first been identified in the cell line FT-MZ-1 established in our laboratory (Runnebaum *et al.*, 1994*b*). We tested primary FTCs for *p53* gene mutation and aberrant protein accumulation, integration of human papillomavirus (HPV) sequences and the association of cell cycle parameters with p53 alterations.

### Material and methods

Pretreatment tumour samples from seven German Caucasian patients diagnosed with primary fallopian tube adenocarcinoma between 1986 and 1993 were analysed. Abdominal hysterectomy with bilateral salpingo-oophorectomy, resection of the omentum and retroperitoneal lymphadenectomy were performed. The tumour specimens contained more than 90% tumour cells as examined on haematoxylin and eosin stained sections taken from the same tumour block preceding and subsequent to the segment analysed. Tissue sections were analysed by two pathologists.

Mutation screening of the entire coding region comprising exon 2 to 11 was carried out using genomic tumour DNA extracted from the paraffin-embedded formalin-fixed sections as described previously (Runnebaum *et al.*, 1991, 1994*b*). Polymerase chain reaction (PCR) fragments were sequenced on an automated sequencer (ALF express, Pharmacia Biotech, Uppsala, Sweden). Sense and antisense strands were analysed three times each. Ovarian cancer cell lines with characterised *p53* mutations served as positive controls. The p53 protein was detected by immunohistochemistry with the anti-(human)p53 mouse monoclonal antibody DO-1 (Dianova, Hamburg, Germany). Cell line FT-MZ-1, with a point missense mutation in codon 175 of the *p53* gene, served as a control for positive staining (Runnebaum *et al.*, 1994*b*). The evaluation was performed by two investigators unaware of the results of the molecular analysis. An immunoreactive score (IRS, see Table II) was assessed for recording staining intensity and proportion of stained cells (Remmele and Stegner 1987; Runnebaum *et al.*, 1996).

Genital-type HPV sequences were screened for using an L1 gene consensus primer PCR and dot blot analysis as well as an E6 gene multiplex PCR (Runnebaum *et al.*, 1995*c*). Genomic DNA from the cervical cancer cell lines HeLa (10 to 50 integrated copies of HPV 18 per genome), SiHa (one to two integrated copies of HPV 16) and CaSki (500 to 600 integrated copies of HPV 16) cells served as positive controls.

Flow cytometric analysis (Becton-Dickinson) was carried out using sections of 30 μm. The samples were coded and the experiments were carried out in a blinded fashion to eliminate observer bias.

### Results

Seven patients with fallopian tube adenocarcinoma were included in the study. Four patients had stage IIA disease (Table I), according to the staging classification of the International Federation of Gynaecologists and Obstetricians (FIGO) with extension to the uterus or the ovaries (Nordin, 1994). Three other patients had a FIGO stage IIIC (Table I)

**Table I** Patient and tumour characteristics

| *Tumour DNA no.* | *Patient's age (years)* | *FIGO stage* | *Grade* | *Histology* |
|---|---|---|---|---|
| 339 | 52 | IIA | 3 | Papillary |
| 459 | 57 | IIIC | 3 | Papillary |
| 777 | 65 | IIIC | 3 | Papillary |
| 906 | 67 | IIA | 2 | Papillary |
| 1417 | 56 | IIA | 2 | Papillary |
| 1773 | 69 | IIA | 3 | Papillary |
| 6097 | 52 | IIIC | 3 | Papillary |

Correspondence: IB Runnebaum, Department of Obstetrics and Gynaecology, Frauenklinik, University of Ulm, Prittwitzstr. 43, 89075 Ulm, Germany. e-mail: ingo.runnebaum@medizin.uni-ulm.de
Received 20 December 1995; revised 15 April 1996; accepted 8 May 1996

**Table II** p53 alterations

| Tumour DNA no. | Mutation in codon | Nucleotide sequence | Mutation type | Heterozygosity | Amino acid substitution | Immunoreactive score[a] |
|---|---|---|---|---|---|---|
| 339 | 277 | TGT to TAT | TI[b] | Yes | Cys to Tyr | 2 |
| 459 | | Wild-type | | | | 0 |
| 777 | 194 | CTT to CT | DEL[c] | Yes | Frame shift | 8 |
| 777 | 267 | CGG to CGA | TI | Yes | Silent | |
| 906 | 285 | GAG to AAG | TI | No | Glu to Lys | 12 |
| 1417 | | Wild-type | | | | 2 |
| 1773 | | Wild-type | | | | 0 |
| 6097 | 175 | CGC to CAC | TI | No | Arg to His | 12 |

[a]The immunoreactive score (IRS) records intensity and proportion of stained cells by multiplication of the proportion score PP (PP = 0, no staining; PP = 1, staining in < 10% of cells; PP = 2, staining in 10–50% of cells; PP = 3, staining in 51–80% of cells; PP = 4, staining in > 80% of cells) and the staining intensity score SI (SI = 0, no staining; SI = 1, weak staining; SI = 2, marked staining; SI = 3, strong staining). [b]TI, transition mutation. [c]DEL, deletion mutation.

of the disease with intraabdominal spread. Tumour no. 777 had metastasised to paraaortic lymph nodes.

Five mutations were identified by nucleotide sequencing (Table II). Two mutations were identified in tumour no. 777, one silent point mutation in exon 8 not leading to an amino acid change and one single base deletion in exon 6 shifting the reading frame (Figure 1). The presence of mutant (mt) and wild-type (wt) sequence in nos. 339 and 777 indicated heterozygosity at the *p53* locus.

Immunohistochemistry showed accumulation of p53 protein with a marked stain in three tumours. In tumours 906, with a homozygous point missense mutation in codon 285, and 6097, with the His175 mutant, the strong stain of more than 80% of the tumour cells resulted in an IRS of 12 (Figure 2). Tumour no. 777 showed an intermediate staining intensity in more than 80% of the cells with an IRS of 8 (Figure 2). Two tumours stained weakly with few cells (IRS 2), i.e. tumour no. 339 with an identified *p53* point missense mutation and tumour no. 1417 with wild-type *p53*. Two tumours showed no detectable p53 expression (data not shown).

Integrated HPV sequences were found in none of the FTCs as tested by L1 consensus primer PCR and dot blot. No HPV16-E6 or HPV18-E6 oncogene was detectable by multiplex PCR (data not shown).

The results of the FACScan analysis of the cell cycle distribution are summarised in Table III. The percentage of cells in S-phase varied between 7.06% and 33.90% in the different tumour samples. The S-phase fraction of tumours with mutant *p53* was 25.74% (median) and of tumours with wild-type *p53*, 12.55% (median). The $G_1/G_0$ fractions varied between 58.84% and 82.60%. The median $G_1/G_0$ fraction of mutant *p53* samples was 62.82% (median) and of wild-type *p53* samples, 82.48% (median). All FTC samples were diploid.

**Figure 1** Representative nucleotide sequence readings in sense direction of the p53 gene of three fallopian tube carcinomas. The arrowheads indicate the position of the missense of frameshift mutation.

### Discussion

The majority of FTCs are adenocarcinomas, stages II or III (Eddy *et al.*, 1984; Rosen *et al.*, 1993; Lacy *et al.*, 1995). The tumours in this study were papillary adenocarcinomas representative for FTC. The tumours were metastatic at the time of diagnosis with FIGO stages IIA and IIIC (Nordin, 1994). In four out of seven FTCs, mutations in the *p53* tumour-suppressor gene were identified. The four point mutations were transition mutations, three of which changed the primary amino acid sequence. *p53* transition mutations have frequently been found in other carcinoma types such as ovarian cancer, breast cancer and colon cancer and are considered to occur spontaneously. The point mutation in tumour no. 777 occurred at the third base position of codon 267 as a silent mutation. In the same tumour, an additional *p53* mutation was found. The single base deletion in codon 194 leads to a frame shift. Small deletions could be caused by DNA replication errors and appear to occur relatively frequently in ovarian cancer (Okamoto *et al.*, 1991; Milner *et al.*, 1993; Runnebaum *et al.*, 1994*a*). The loss of heterozygosity (LOH) rate at the *p53* gene locus on chromosome arm 17p is not known for FTC. In various tumours the rate of LOH at this locus exceeds the rate of *p53* mutations. Two out of four FTCs with mutant *p53*, however, remained heterozygous at the *p53* locus.

The identified mutations were located in the 'core region', which comprises codons 102 to 292 of the *p53* gene (Cho *et al.*, 1994). The core region conveys the sequence-specific DNA-binding activity, a key function for the biological effect of p53. Missense mutations in this region are commonly

**Figure 2** Immunohistochemical analysis of p53 expression in fallopian tube carcinomas. (**a**) No. 6097, DO-1. (**b**) No. 6097, negative control. (**c**) No. 777, DO-1. (**d**) No. 777, negative control. Size bar = 20 $\mu$m.

reflected by a nuclear accumulation of p53 protein stabilised in a denatured state (Cho *et al.*, 1994). Studies have been conducted based on immunohistochemical screening to establish accumulation of p53 protein as a marker of prognostic significance in diverse tumour types. Accumulation of p53 as studied in 43 FTC cases has not been found to

**Table III** p53 status and cell cycle parameters

| *Tumour no.* | *p53 status* | *$G_0/G_1$ (%)* | *S (%)* | *$G_2/M$ (%)* |
|---|---|---|---|---|
| 339 | Mutant | 71.33 | 24.44 | 4.23 |
| 777 | Mutant | 64.48 | 23.12 | 12.40 |
| 906 | Mutant | 61.16 | 33.90 | 4.94 |
| 6097 | Mutant | 58.84 | 27.03 | 14.13 |
| 459 | Wild-type | 82.60 | 12.55 | 4.85 |
| 1417 | Wild-type | 65.49 | 16.37 | 18.14 |
| 1773 | Wild-type | 82.48 | 7.06 | 10.46 |

predict clinical outcome (Lacy *et al.*, 1995); the staining intensity but not the fraction of stained cells was recorded. Superior to the assessment of staining intensity or percentage of stained cells alone could be the IRS as demonstrated in a study on the prognostic value of steroid receptor expression in ovarian cancer (Kieback *et al.*, 1993). Not all point missense mutations lead to accumulation of denatured p53 protein which was observed in one out of four tumours with *p53* missense mutations in our study. An immunohistochemical study without molecular analysis may therefore mistakenly rate a significant number of tumours as containing wild-type p53. It appears difficult to value p53 immunohistochemical data with regard to clinical outcome.

Binding of p53 to the HPV E6 oncogene product mediated by the E6-associated protein has been shown to be a mechanism of p53 inactivation in squamous cell carcinoma of the cervix. Genital-type HPV sequences were not detected in the seven FTC samples analysed. HPV-related p53 inactivation (Scheffner *et al.*, 1990) may not play a role in the development of FTC.

FTCs with mutant *p53* presented with a higher S-phase, even in the p53 heterozygous tumours. Wild-type p53 protein, increased by DNA damage, transcriptionally induces $p21^{WAF1/CIP1}$ (El-Deiry *et al.*, 1993; Harper *et al.*, 1993). $p21^{WAF1/CIP1}$ complexes with and inhibits factors essential for cell cycle progression at the $G_1$ checkpoint and DNA replication, cyclin-dependent kinases and the proliferating cell nuclear antigen (PCNA) (Chen *et al.*, 1995; Luo *et al.*, 1995). In the *p53* heterozygous tumour no. 339, wild-type p53 may be inactivated in the presence of a dominant negative mutant protein possibly sequestering the wild-type protein by oligomerisation (Clore *et al.*, 1994).

In a small number of fallopian tube cancers, we have demonstrated that the *p53* tumour-suppressor gene can be mutated with accumulation of aberrant p53 protein. FTCs with a *p53* mutation may present with a higher S and a lower $G_1/G_0$ fraction. Larger studies will define the role of *p53* mutation and cell cycle parameters as indicators of clinical outcome. Because of the rarity of FTC, such studies could best be performed in multicentre cooperative study groups.

**Acknowledgements**
The authors wish to thank Ms Dreher for excellent technical help in FACS analysis and Sabine Maier for HPV analysis. This work was supported in part by the Deutsche Forschungsgemeinschaft (DFG RU476/2-1) and institutional awards (P.119/1993 and P.234/95, Forschungsförderungsprogramm des Klinikumsvorstandes der Universität Ulm) granted to IBR.

### References

BAKER SJ, MARKOWITZ S, FEARON ER, WILLSON JK AND VOGELSTEIN B. (1990). Suppression of human colorectal carcinoma cell growth by wild-type p53. *Science*, **249,** 912–915.

CHEN J, JACKSON PK, KIRSCHNER MW AND DUTTA A. (1995). Separate domains of p21 involved in the inhibition of Cdk kinase and PCNA. *Nature*, **374,** 386–388.

CHENG J, YEE JK, YEARGIN J, FRIEDMANN T AND HAAS M. (1992). Suppression of acute lymphoblastic leukemia by the human wild-type p53 gene. *Cancer Res.*, **52,** 222–226.

CHO Y, GORINA S, JEFFREY PD AND PAVLETICH NP. (1994). Crystal structure of a p53 tumor suppressor-DNA complex: Understanding tumorigenic mutations. *Science*, **265,** 346–355.

CLORE GM, OMICHINSKI JG, SAKAGUCHI K, ZAMBRANO N, SAKAMOTO H, APPELLA E AND GRONENBORN AM. (1994). High-resolution structure of the oligomerization domain of p53 by multidimensional NMR. *Science*, **265,** 386–391.

EDDY GL, COPELAND LJ, GERSHENSON DM, ATKINSON EN, WHARTON JT AND RUTLEDGE FN. (1984). Fallopian tube cacinoma. *Obstet. Gynecol.*, **64,** 546–552.

EL-DEIRY WS, TAKINO T, VELCULESCU VE, LEVY DB, PARSON R, TRENT JM, LIN D, MERCER WE, KINZLER KW AND VOGELSTEIN B. (1993). WAF1, a potential mediator of p53 tumor suppression. *Cell*, **75,** 817–825.

ELIYAHU D, MICHALOVITZ D, ELIYAHU S, PINHASI KO AND OREN M. (1989). Wild-type p53 can inhibit oncogene-mediated focus formation. *Proc. Natl Acad. Sci. USA*, **86,** 8763–8767.

HARPER JW, ADAMI GR, WEI N, KEYOMARSI K AND ELLEDGE SJ. (1993). The p21 cdk-interacting protein Cip1 is a potent inhibitor of G1 cyclin-dependent kinases. *Cell*, **75,** 805–816.

KASTAN MB, ONYEKWERE O, SIDRANSKY D, VOGELSTEIN B AND CRAIG RW. (1991). Participation of p53 protein in the cellular response to DNA damage. *Cancer Res.*, **51,** 6304–6311.

KASTAN MB, ZHAN Q, EL-DEIRY WS, CARRIER F, JACKS T, WALSH WV, PLUNKETT BS, VOGELSTEIN B AND FORNACE AJJ. (1992). A mammalian cell cycle checkpoint pathway utilizing p53 and GADD45 is defective in ataxia-telangiectasia. *Cell*, **71,** 587–597.

KERN SE, KINZLER KW, BRUSKIN A, JAROSZ D, FRIEDMAN P, PRIVES C AND VOGELSTEIN B. (1991). Identification of p53 as a sequence-specific DNA-binding protein. *Science*, **252,** 1708–1711.

KIEBACK DG, PRESS MF, ATKINSON EN, MÖBUS VJ, RUNNEBAUM IB, KREIENBERG R AND JONES LA. (1993). Prognostic significance of estrogen receptor expression in ovarian cancer. Immunoreactive score (IRS) vs. composition adjusted receptor level (CARL). *Anticancer Res.*, **13,** 2489–2496.

KUPRYJANCZYK J, THOR AD, BEAUCHAMP R, MERRITT V, EDGERTON SM, BELL DA AND YANDELL DW. (1993). p53 gene mutations and protein accumulation in human ovarian cancer. *Proc. Natl Acad. Sci. USA*, **90,** 4961–4965.

LACY MQ, HARTMANN LC, KEENEY GL, CHA SC, WIEAND HS, PODRATZ KC AND ROCHE PC. (1995). c-erbB-2 and p53 expression in fallopian tube carcinoma. *Cancer*, **75,** 2891–2896.

LOWE SW AND RULEY HE. (1993). Stabilization of the p53 tumor suppressor is induced by adenovirus 5 E1A and accompanies apoptosis. *Genes Dev.*, **7,** 535–545.

LUO Y, HURWITZ J AND MASSAGUE J. (1995). Cell-cycle inhibition by independent CDK and PCNA binding domains in p21Cip1. *Nature*, **375,** 159–161.

MARKS JR, DAVIDOFF AM, KERNS BJ, HUMPHREY PA, PENCE JC, DODGE RK, CLARKE-PEARSON D, IGLEHART JD, BAST RCJ AND BERCHUCK A. (1991). Overexpression and mutation of p53 in epithelial ovarian cancer. *Cancer Res.*, **51,** 2979–2984.

MILNER BJ, ALLAN LA, ECCLES DM, KITCHENER HC, LEONARD RCF, KELLY KF, PARKIN DE AND HAITES NE. (1993). p53 Mutation is a common genetic event in ovarian carcinoma. *Cancer Res.*, **53,** 2128–2132.

MIYASHITA T, KRAJEWSKI S, KRAJEWSKA M, WANG HG, LIN HK, LIEBERMANN DA, HOFFMAN B AND REED JC. (1994). Tumor suppressor p53 is a regulator of bcl-2 and bax gene expression *in vitro* and *in vivo*. *Oncogene*, **9,** 1799–1805.

MIYASHITA T AND REED JC. (1995). Tumor suppressor p53 is a direct transcriptional activator of the human bax gene. *Cell*, **80,** 293–299.

NORDIN AJ. (1994). Primary carcinoma of the fallopian tube: a 20-year literature review. *Obstet. Gynecol. Surv.*, **49,** 349–361.

OKAMOTO A, SAMESHIMA Y, YOKOYAMA S, TERASHIMA Y, SUGIMURA T, TERADA M AND YOKOTA J. (1991). Frequent allelic losses and mutations of the p53 gene in human ovarian cancer. *Cancer Res.*, **51,** 5171–5176.

REMMELE W AND STEGNER HE. (1987). Vorschlag zur einheitlichen Definition eines Immunreaktiven Score (IRS) für den immunhistochemischen Östrogenrezeptor-Nachweis. *Pathologe*, **8,** 138–140.

ROSE PG, PIVER MS AND TSUKADA Y. (1990). Fallopian tube cancer. The Roswell Park experience. *Cancer*, **66,** 2661–2667.

ROSEN A, KLEIN M, LAHOUSEN M, GRAF AH, RAINER A AND VAVRA N. (1993). Primary carcinoma of the fallopian tube – a retrospective analysis of 115 patients. Austrian Cooperative Study Group for Fallopian Tube Carcinoma. *Br. J. Cancer*, **68,** 605–609.

RUNNEBAUM IB AND KREIENBERG R. (1995). p53 trans-dominantly suppresses tumor formation of human breast cancer cells mediated by retroviral bulk infection without marker gene selection: an expeditious *in vitro* protocol with implications towards gene therapy. *Hybridoma*, **14,** 153–157.

RUNNEBAUM IB, NAGARAJAN M, BOWMAN M, SOTO D AND SUKUMAR S. (1991). Mutations in p53 as potential molecular markers for human breast cancer. *Proc. Natl Acad. Sci. USA*, **88,** 10657–10661.

RUNNEBAUM IB, TONG X-W, MOEBUS V, HEILMANN V, KIEBACK DG AND KREIENBERG R. (1994*a*). Multiplex PCR (MPCR) screening detects small p53 deletions and insertions in human ovarian cancer cell lines. *Hum. Genet.*, **93,** 620–624.

RUNNEBAUM IB, TONG XW, MÖBUS VJ, KIEBACK DG, ROSENTHAL HE AND KREIENBERG R. (1994*b*). p53 mutant His175 identified in a newly established fallopian tube carcinoma cell line secreting interleukin 6. *FEBS Lett.*, **353,** 29–32.

RUNNEBAUM IB, YEE J-K, KIEBACK DG, SUKUMAR S AND FRIEDMANN T. (1994*c*). Wild-type p53 suppresses the malignant phenotype in breast cancer cells containing mutant p53 alleles. *Anticancer Res.*, **14,** 1137–1144.

RUNNEBAUM IB, TONG XW, KÖNIG R, HONG Z, KÖRNER K, ATKINSON EN, KREIENBERG R AND KIEBACK DG. (1995*a*). p53-based blood test for *p53PIN3* and risk for sporadic ovarian cancer. *Lancet*, **345,** 994.

RUNNEBAUM IB, WANG S AND KREIENBERG R. (1995*b*). Retrovirally mediated wild-type p53 restores S phase modulation without inducing *WAF*1 mRNA in breast carcinoma cells containing mutant p53. *J. Cell. Biochem.*, **59,** 538–544.

RUNNEBAUM IB, MAIER S, TONG XW, ROSENTHAL HE, MÖBUS VJ, KIEBACK DG AND KREIENBERG R. (1995*c*). Human papillomavirus integration is not associated with advanced epithelial ovarian cancer in German patients. *Cancer Epidemiol. Biomarkers*, **4,** 573–575.

RUNNEBAUM IB, KIEBACK DG, MÖBUS VJ, TONG X-W AND KREIENBERG R. (1996). Subcellular localization of accumulated p53 in ovarian cancer cells. *Gynecol. Oncol.*, **61,** 266–271.

SCHEFFNER M, WERNESS BA, HUIBREGTSE JM, LEVINE AJ AND HOWLEY PM. (1990). The E6 oncoprotein encoded by human papillomavirus types 16 and 18 promotes the degradation of p53. *Cell*, **63,** 1129–1136.

TSAO SW, MOK CH, OIKE K, MUTO M, GOODMAN HM, SHEETS EE, BERKOWITZ RS, KNAPP RC AND LAU CC. (1991). Involvement of p53 gene in the allelic deletion of chromosome 17p in human ovarian tumors. *Anticancer Res.*, **11,** 1975–1982.

UNGER T, NAU MM, SEGAL S AND MINNA JD. (1992). p53: a transdominant regulator of transcription whose function is ablated by mutations occurring in human cancer. *Embo J.*, **11,** 1383–1390.

YONISH-ROUACH E, GRUNWALD D, WILDER S, KIMCHI A, MAY E, LAWRENCE J-J, MAY P AND OREN M. (1993). p53-mediated cell death: relationship to cell cycle control. *Mol. Cell. Biol.*, **13,** 1415–1423.